# Quadruple-negative breast cancer: novel implications for a new disease

**DOI:** 10.1186/s13058-020-01369-5

**Published:** 2020-11-19

**Authors:** Shristi Bhattarai, Geetanjali Saini, Keerthi Gogineni, Ritu Aneja

**Affiliations:** 1 Department of Biology, Georgia State University, 100 Piedmont Ave, Atlanta, GA 30303 USA; 2 Department of Hematology and Medical Oncology, Emory University School of Medicine, Atlanta, GA 30322 USA

**Keywords:** Quadruple-negative breast cancer, Triple-negative breast cancer, Androgen receptor, AR antagonists, Cancer biomarkers, Therapeutic targets

## Abstract

Based on the androgen receptor (AR) expression, triple-negative breast cancer (TNBC) can be subdivided into AR-positive TNBC and AR-negative TNBC, also known as quadruple-negative breast cancer (QNBC). QNBC characterization and treatment is fraught with many challenges. In QNBC, there is a greater paucity of prognostic biomarkers and therapeutic targets than AR-positive TNBC. Although the prognostic role of AR in TNBC remains controversial, many studies revealed that a lack of AR expression confers a more aggressive disease course. Literature characterizing QNBC tumor biology and uncovering novel biomarkers for improved management of the disease remains scarce. In this comprehensive review, we summarize the current QNBC landscape and propose avenues for future research, suggesting potential biomarkers and therapeutic strategies that warrant investigation.

## Introduction

Triple-negative breast cancer (TNBC) is defined by the lack of the estrogen receptor (ER), progesterone receptor (PR), and human epidermal growth factor receptor 2 (HER2) expression [1]. Compared to other breast cancer (BC) subtypes, patients with TNBC have a more aggressive clinical course and poorer prognosis, with higher rates of local recurrence and distant metastasis within 5 years of diagnosis [2]. The disease disproportionately affects women of African ancestry and pre-menopausal women [2–5]. Mounting evidence suggests that non-biological and epidemiological factors play a substantial role in the etiology of TNBC. Factors associated with increased risk of TNBC include African ancestry, younger age, obesity, BMI, shorter breastfeeding duration, higher parity, oral contraceptive usage for ≥ 1 year, low socioeconomic status, a diet high in animal fat, and low physical activity. Some of these, including obesity, breastfeeding, and physical inactivity, have been linked to androgen secretion dysregulation.

The stark inter-patient and intra-tumoral heterogeneity render TNBC challenging to cure [6, 7]. Based on gene expression profiling, TNBCs have been sub-classified, raising the potential for more precise therapeutic intervention.

An updated version by Lehmann and colleagues classifies TNBC into four distinct molecular subtypes including two basal-like subtypes, i.e., (BL1 and BL2), mesenchymal (M), and luminal androgen receptor (LAR) [8]. Retrospective analysis of these TNBC subtypes demonstrated significant differences in response to neoadjuvant chemotherapy between subtypes, with LAR displaying worse response and BL1 responding more favorably [9]. Additionally, Burstein et al. categorize TNBC into luminal androgen receptor (LAR), mesenchymal (MES), basal-like immunosuppressed (BLIS), and basal-like immune activated (BLIA) [10].

Although TNBCs exhibit greater chemosensitivity compared to non-TNBCs, many TNBC patients diagnosed with advanced-stage disease relapse following treatment with conventional anthracycline/taxane-based chemotherapy [3–5]. Hence, the identification of robust prognostic biomarkers and novel therapeutic targets is of high clinical importance.

Androgen receptor (AR) is expressed in approximately 10–43% of TNBCs depending on the AR positivity cutoff used; therefore, it has emerged as a promising therapeutic target for TNBC patients [11]. AR antagonists, such as enzalutamide and bicalutamide, currently in clinical trials, are showing promising results in AR-positive TNBC patients [12–16]. However, the remaining 67%–90% of TNBCs that lack AR expression, also referred to as quadruple-negative breast cancers (QNBCs), do not benefit from AR antagonists, and some studies have reported a worse prognosis for QNBC patients [17–22] compared to those with AR-positive TNBC. Recent evidence suggests that QNBC differs from TNBC in its molecular and genetic make-up. This calls for extensive identification and annotation of key AR-dependent proteins and a delineation of the mechanistic action of AR. Herein, we summarize the current landscape of QNBC, implications of AR absence or presence, and rationally designed therapeutic strategies for QNBCs, as well as suggest avenues for future research in disease management.

## Cutting both ways: the role of AR in TNBC

AR is a transcription factor (TF) belonging to the nuclear steroid hormone receptor family. AR-mediated signaling plays a critical role in the development of breast tissue [23]. There is also accumulating evidence supporting the role of AR in BC development and progression [24, 25]. Nonetheless, the role of AR signaling in TNBC remains unclear. In TNBC, AR is reported to interact with androgen response elements (AREs) and stimulate tumor cell growth in an androgen-dependent manner. In TNBC patients, clinical trial focused on testing the anti-androgen therapies (e.g., bicalutamide, abiraterone acetate + prednisone, and enzalutamide) in combination with chemotherapy in TNBC patients have yielded positive results [15, 16, 26]. Currently, the efficacy of various drug combinations such as pembrolizumab and enobosarm, palbociclib and avelumab, and taselisib and enzalutamide is being evaluated in patients with metastatic TNBC [27–30]. In addition, the ongoing MDACC ARTEMIS trial is a neoadjuvant study that is focused on women with stage I–III TNBC and modifies therapy based on molecular profiling. For example, if AR is expressed, then enzalutamide along with paclitaxel is administered [31].

There have been conflicting reports regarding AR expression levels in TNBC, ranging from 7 to 75% [32–36]. In contrast to non-LAR subtypes, the TNBC LAR subtype is enriched in AR expression and exhibits sensitivity to AR-targeted therapies [37]. However, the prognostic role of AR in TNBC is ambiguous, with several studies associating loss of AR expression with worse prognosis in TNBC patients, and others attributing worse outcomes to increased AR signaling [14, 19–21, 33–35, 38–48]. These discrepancies are largely attributed to variability in sample procuring methods, antibodies, staining, scoring methods, and AR positivity cutoff values. The confounding effects of patient selection in prospective studies could also be a plausible factor underlying this variability [49]. We have recently conducted a multi-institutional study evaluating AR expression among different cohorts and observed that, even after accounting for these factors, the discrepancy in prognostic value persisted [50].

Additionally, AR splice variants (AR-Vs), which are produced due to structural rearrangement or alternative splicing of the AR transcript, have been suggested to underlie the variability in findings regarding the prognostic role of AR in TNBC [51]. These variants lack the entire or a part of the ligand-binding domain (LBD; the target of enzalutamide) yet remain constitutively active and activate target genes [51, 52]. Thus far, 15 different AR-Vs have been identified [53, 54]. In prostate cancer, the expression of AR-Vs has been shown to confer resistance to androgen deprivation therapy [55, 56]. In a recent study, Hickey et al. [57] reported the expression of active AR-Vs in ER-negative breast tumors, suggesting their potential influence in the response of TNBCs to androgen deprivation therapies. The study also highlighted the role of the well-studied AR-V7 in inducing in vitro cell proliferation in the absence of enzalutamide. AR and AR-V7 differentially regulate target gene expression depending on the preferential recruitment of AR or its splice variant to specific *cis*-regulatory DNA sequences [58]. AR-V7 expression has recently been reported to be associated with unfavorable BC patient prognosis, which has increased interest in investigating this AR-V as a potential therapeutic target [11, 59]. Limited data on AR-V7 and other AR-Vs exists in BC; thus, further investigation into the different AR-Vs may help resolve the controversial prognostic role of AR in TNBC patients.

AR can drive tumor growth even in TNBC subtypes expressing low levels of AR. Although the precise mechanisms remain unclear, the AR-positive tumor cell subpopulation can support the growth of a cancer stem cell (CSC)-like cells, which promote chemotherapy resistance and tumor recurrence. Thus, AR-targeting agents may still be beneficial for TNBC patients exhibiting low (≤ 1%) AR expression [60]. Compared with paclitaxel alone, the combination of enzalutamide and paclitaxel is believed to be more effective in preventing recurrence by targeting CSC-like cells.

Augmenting the complexity of the subject further, prognostic implications of AR expression appear to vary by ancestry. A recent AR assessment study [61] found that TNBC in women of African ancestry (AA) was associated with a higher frequency of AR expression loss. Furthermore, AAs with AR-negative TNBC had worse overall survival than women of European ancestry (EA). AA women with AR-negative TNBC were found to express a unique molecular signature and were enriched for BL1, BL2, and IM subtypes.

**Fig. 1:**
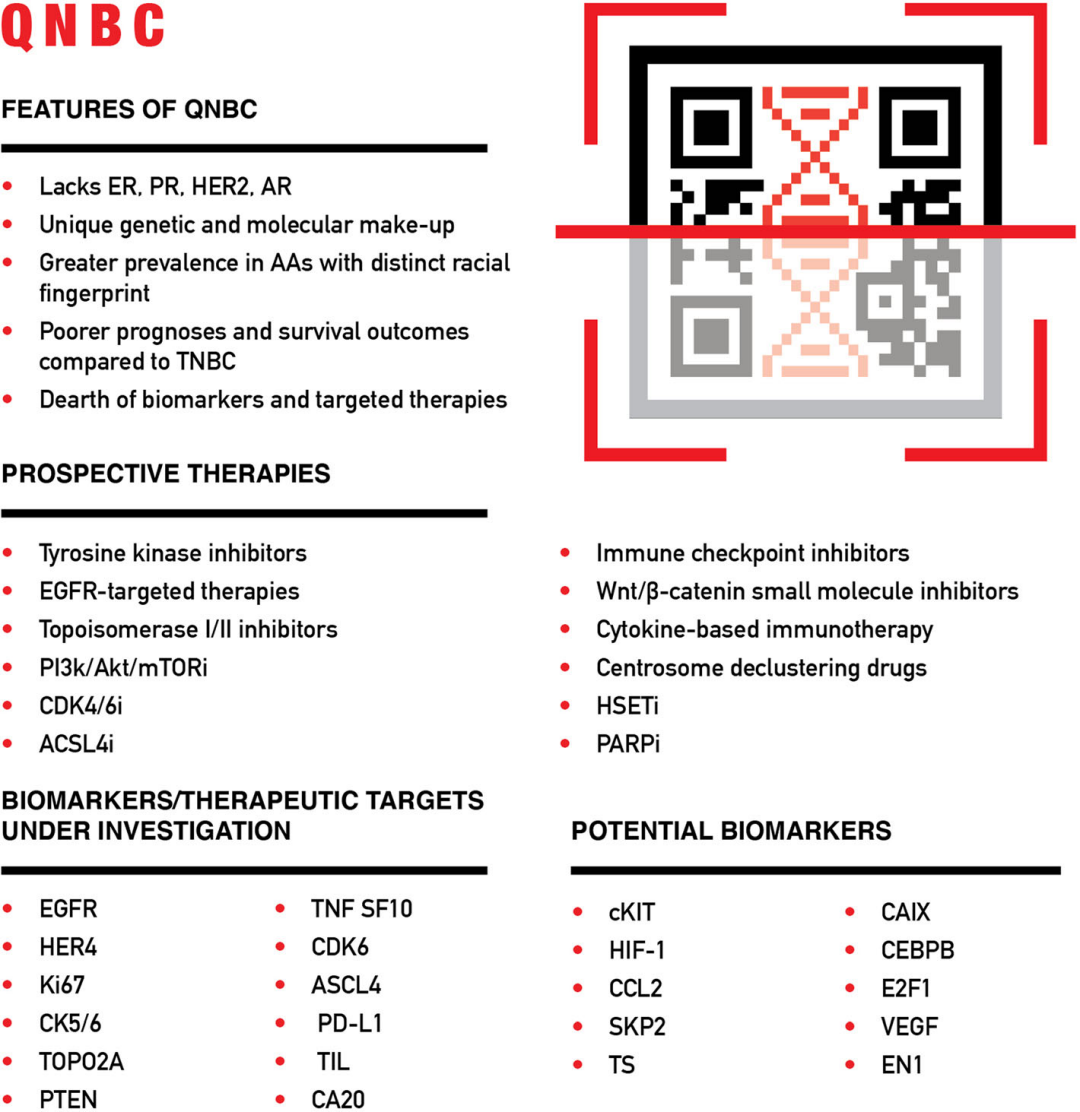
Overview of the distinct features of QNBC as well as biomarkers/therapeutic targets and therapies under investigation. QNBC is clustered with the TNBC subtype despite having a unique molecular landscape. QNBC warrants an in-depth annotation and should be considered a separate BC subtype

In contrast to ER, PR, and HER2 testing, which is standard clinical practice, AR testing has not been standardized due to the lack of consensus regarding its prognostic value. However, given expanding data revealing that QNBC tumors have a more aggressive disease course and worse outcomes than AR-positive tumors, standardizing AR testing is critical. QNBC has a distinct molecular profile and should be considered a separate entity from TNBC. TNBC heterogeneity is associated with AR expression, suggesting that QNBC is a distinct, clinically relevant subtype [62]. Differences in tumor biology between QNBC and TNBC can be exploited to yield novel therapies for targeted management of AR-negative and AR-positive TNBCs. Table 1 captures these distinctions and corresponding potential therapeutic interventions for AR-negative TNBC patients (also see Fig. 1).

**Table 1:** QNBC biomarkers and therapeutic targets, based upon differences in tumor biology between AR-negative and AR-positive TNBCs, and suggested therapeutic interventions

QNBC biomarkers and therapeutic targets	Genomic and molecular features (relative expression to AR+)	Prospective therapy
*Cell growth and proliferation*		
EGFR (epidermal growth factor receptor)	Higher expression; indicates increased cellular growth and proliferation	Tyrosine kinase inhibitors (gefitinib and erlotinib) and anti-EGFR monoclonal antibodies (cetuximab) [63–67]
HER4 (human epidermal growth factor receptor 4)	Lack of expression; may serve as a prognostic biomarker	Not a therapeutic target; only a prognostic biomarker [68–70]
Ki-67	Enhanced expression, i.e., high proliferation index	Anthracycline/taxane-based chemotherapy [12, 71–76]
CK 5/6 (*cytokeratin 5/6*)	Enhanced expression	
TOPO2A (topoisomerase IIα)	Elevated levels	Anthracycline, topoisomerase I/II inhibitors and PI3K/AKT/mTOR inhibitors [77, 78]
PTEN (phosphatase and tensin)	Decreased expression	
CDK6 (cyclin-dependent kinase 6)	Increased mRNA expression	CDK4/6 inhibitors (palbociclib, trilaciclib) [79, 80]
*Cell metabolism*		
ASCL4 (acyl-CoA synthetase 4)	- Elevated expression associated with claudin-low and basal-like BC phenotypes; may boost arachidonic acid metabolism through PTGS2, ALOX5, and AKT/mTOR pathways	- Synergistic effect of ACSL4 inhibitor (e.g., rosiglitazone) and mTOR inhibitor (e.g., rapamycin)
	- May serve as a therapeutic biomarker	- Downregulation of ASCL4 upregulates ER and AR expression in vitro; ASCL4 inhibition may create sensitivity to hormone-targeted therapies such as tamoxifen and anti-AR agents [81–85]
*Tumor immune microenvironment*		
PD-L1 (programmed death-ligand 1)	Higher expression	Immune checkpoint inhibitors (i.e., pembrolizumab) [86]
TIL (tumor-infiltrating lymphocytes)	Higher peripheral and stromal levels (suggests increased anti-tumor immune activity); positively correlates with EGFR, BRCA1, β-catenin expression in early-stage QNBC	EGFR-targeted therapies, platinum agents, and Wnt/β-catenin small molecule inhibitors [87–89]
TNFSF10 (tumor necrosis factor superfamily member 10)	Lower mRNA expression (suggests decreased anti-tumor immune activity)	Potential susceptibility to cytokine-based immunotherapy to stimulate anti-tumoral immunity [68, 90]
*Organellular level*		
CA20 gene set	Higher centrosome amplification (CA) and CA20 score	Centrosome declustering drugs (griseofulvin, noscapine), HSET inhibitors (CW069, AZ82), PARPi (PJ34, GF-15) [91–99]

Although the prognostic value of AR is ambiguous, there is better agreement on its predictive value. A study by Masuda et al. [100] demonstrated that among TNBC subtypes, BL1 had the highest pathological complete response (pCR) rate (52%) after neoadjuvant chemotherapy (NAC), while BL2 and LAR had the lowest. Low AR levels were associated with higher pCR rates in a clinical trial evaluating neoadjuvant cisplatin plus paclitaxel with or without everolimus in a TNBC cohort [101]. Another study found that AR could predict tamoxifen treatment benefit in TNBC patients, where AR-positive patients benefited from the treatment, whereas patients with AR-negative tumors progressed after tamoxifen treatment [102]. Altogether, AR status testing can aid in neoadjuvant treatment decision-making.

## In-depth mapping of the QNBC terrain: unearthing novel targets

Protein expression analysis, gene copy number analysis, and gene sequencing have yielded promising therapeutic targets for TNBC (Fig. 1). A recent integrated network analysis and machine learning approach identified key genes and pathways to help distinguish TNBCs from non-TNBCs [103]. In contrast, genome wide studies examining differences between AR-positive TNBCs and AR-negative QNBCs are extremely scarce. QNBCs are not yet explicitly classified as a separate BC subtype and are overshadowed by the greater focus that TNBC research receives. In addition, within TNBCs, more AA women tend to be AR-negative/QNBC, a demographic that is persistently under-represented in clinical studies and public datasets such as TCGA. In this section, we review the limited available data exploring potential biomarkers in QNBC that may serve as therapeutic targets. Further study of whether druggable targets overexpressed in TNBC are also overexpressed in QNBC is warranted. Results from the Caris Research Institute corroborated higher expression of EGFR and TOPO2A in QNBC compared to AR-positive TNBCs, as well as indicated significantly higher expression of c-KIT and thymidylate synthase (TS) [104]. C-Kit is a receptor tyrosine kinase (RTK), and an increased gene copy number has been linked to an aggressive phenotype and unfavorable prognosis in TNBC [105]. Jansson et al. [106] also observed higher protein levels of c-KIT and two other RTKs, vascular endothelial growth factor receptor-2 (VEGFR2), and platelet-derived growth factor receptor alpha (PDGFRα), in TNBC compared to non-TNBC [107–110]. Specimens obtained in trials targeting these markers in TNBC should be interrogated for AR status and whether response varies by AR status. Small molecule inhibitors and monoclonal antibodies are the main RTK-targeting therapeutic approaches under development in TNBC. Both FDA-approved and investigational drugs such as imatinib, cabozantinib, dasatinib, lucitanib, and sunitinib (inhibits kinases and/or VEGFR, FGFR, PDGFR) are being evaluated in TNBC patients [107–111] and could be of value in QNBCs as well. TS catalyzes the conversion of deoxyuridine monophosphate (dUMP) to deoxythymidine monophosphate (dTMP) and is involved in nucleotide metabolism, which is often boosted in cancer cells to sustain increased proliferation. A 2019 study reported significantly high levels of TS in TNBC compared to luminal BC subtypes, which correlated with worse prognosis and could prove to be a valuable prognostic biomarker and therapeutic target in QNBC [112].

Davis et al. [61] identified gene expression differences in AR-deficient versus AR-expressing BC patients by race, providing a template to advance QNBC research. A comprehensive assessment of AR expression within all BC subtypes from The Cancer Genome Atlas (TCGA) revealed that women of AA were more likely to lack AR expression than White women. The association between AR expression and race was strongest in TNBC. These findings highlight the need for extensive race-specific annotation of genes associated with AR-negative BC. Further examination of AR-negative BCs for a distinctive gene expression profile has uncovered differential expression of several immune-related genes by race, including *E2F1*, *PDK1*, *CCL2*, *CEBPB*, *NFKBIL2*, *TGFB3*, *IL12RB2*, *IL2RA*, and *SOS1* (Fig. 1).

### E2F1 and angiogenesis markers

E2F1 belongs to a class of TFs involved in G1/S transition and is being touted as a master regulator of BC metastasis [113]. Non-steroidal anti-inflammatory drugs such as diclofenac and indomethacin were found to downregulate E2F1, among other genes, and inhibit cell growth in ovarian cancer cell lines [114]. E2F1 affects the activity of several key signaling pathways, including hypoxia and angiogenesis, both of which are considered hallmarks of cancer [113]. E2F1 modulates angiogenesis via vascular endothelial growth factor (VEGF), its key facilitator [115, 116]. A significantly higher expression of VEGF has been observed in TNBCs compared to non-TNBCs [117]. Nevertheless, the role of angiogenesis in QNBC remains elusive. VEGF signaling is reportedly upregulated by EGFR expression, which is also significantly higher among QNBCs compared to AR+ TNBCs [118]. Clinical trials testing VEGF inhibitors, such as bevacizumab, in combination with cytotoxic chemotherapy or other antiangiogenic agents in metastatic BC and TNBC patients, have shown an improved patient response [119]. Extensive studies assessing the role of other key angiogenesis markers in addition to VEGF, such as mTOR, fibroblast growth factor (FGF), Notch, hypoxic-inducible factor (HIF), and insulin growth factor (IGF), as potential novel therapeutic targets in QNBC are required.

### Mediators of hypoxia

Interestingly, mediators of hypoxia, such as HIF TFs (HIF-1α and HIF2α) and carbonic anhydrase IX (CAIX), are highly upregulated in TNBCs compared to non-TNBCs [120, 121]. Moreover, high CAIX expression has been associated with poor disease-free survival (DFS) and overall survival (OS) among AR-negative/ER-negative BC patients [122]. HIF1-α downregulation in TNBC cells promoted apoptosis and impaired cell invasion and migration, while CAIX silencing in metastatic BC mouse models resulted in regression of orthotopic mammary tumors. Hence, it may be useful to investigate differences in hypoxia-induced proteins between AR-negative and AR-positive TNBCs to determine whether targeting HIF1-α and CAIX will be beneficial in QNBC [123, 124]. HIF-1 and CAIX small molecule inhibitors and monoclonal antibodies are currently in preclinical and early clinical testing [123, 125].

### PDK1

One of the differentially expressed genes in QNBC identified by Davis et al. is 3-phosphoinositide-dependent protein kinase-1 (PDK1), a downstream effector of PI3K [61]. The role of PDK in carcinogenesis is suggested to be PIK3/AKT pathway-dependent; however, in BC, PDK1 is also believed to be activated in a PIK3/AKT-independent manner [126, 127]. PDK1 depletion has been shown to delay tumor initiation, progression, and metastasis in a BC mouse model [128]. Thus, the PDK1 signaling pathway may represent a viable therapeutic target in QNBC. 2-*O*-Bn-InsP5 (M. Falasca Laboratory), GSK2334470 (GlaxoSmithKline), OXIDs (S. Rapposelli Laboratory), and MP7 (Merck) are some of the PDK1 inhibitors under investigation [129].

### Tumor immune landscape

The chemokine CCL2 is involved in tumor development and progression by promoting the migration and infiltration of monocytes and tumor-associated macrophages (TAMs) [130]. CCL2 overexpression has been associated with poor patient prognosis in various tumor types, including BC [131]. TAMs and tumor-associated neutrophils (TANs) are significantly more prevalent in TNBC than in hormone receptor-positive BC [132–134]. Although TAMs and TANs participate in anti-tumor immunity, they can also switch from a pro-inflammatory cell phenotype to a pro-tumoral one. One of the immunosuppressive functions of TAMs in TNBC is the induction of the co-inhibitory molecules PD-1 and TIM-3 [135]. While CCL2 is a potential molecular target, therapeutic intervention using neutralizing antibodies against CCL2 has not yielded the desired impact. Using a novel gene silencing approach, Fang et al. [131] were able to target CCL2 more effectively, inhibiting TNBC progression by blocking CSC renewal and M2 macrophage recruitment. Therapeutic strategies to suppress the pro-tumoral functions of TAMs and TANs are currently under investigation [136, 137]. Expression of CD4+ and CD8+ T cell markers along with that of the immune checkpoint molecules PD-1, PD-L1, and CTLA-4 was found to be significantly upregulated in QNBC compared with AR-positive TNBC [61]. Therefore, it may be worthwhile to investigate differential expression of other immune checkpoint molecules, such as LAG-3 and TIM-3. In addition to PD-L1 inhibitors, immune checkpoint inhibitors targeting CTLA-4 and LAG-3, such as ipilimumab and IMP321, have reached phase III and I/II clinical trials, respectively, and have shown promising results [138]. A comprehensive elucidation of the QNBC immune landscape could be invaluable for risk prognostication and targeted immunotherapeutic intervention.

### Micro-RNAs

Shi et al. [139] identified 153 micro-RNAs (miRNAs) that were differentially expressed between QNBC and AR-positive molecular subtypes, affecting several signaling pathways involved in tumor cell proliferation and invasion. Another study using TCGA data identified 40 miRNAs with differential expression in QNBC. Interestingly, these miRNAs were associated with race and BC subtype [62]. Current efforts are focused on the development of therapeutic strategies targeting miRNAs in cancer and other diseases, such as miRNA mimics and anti-miRNAs. The identification of QNBC-specific circulating miRNAs may also improve detection and prognosis.

### Transcription factors: CEBPB and EN1

The CCAAT enhancer-binding protein beta (CEBPB) is a TF regulating the expression of genes involved in inflammatory responses. High CEBPB has been correlated with expression of the chromosome 19 miRNA cluster (C19MC), the expression of which has been linked to TNBC [140]. Due to this association, it may also be worthwhile to compare C19MC expression in QNBC versus TNBC. The fact that CEBPB is induced under hypoxic conditions [141] further emphasizes the need to determine the role of hypoxia in QNBC.

From analyses in TCGA datasets, Peluffo G et al. [142] identified 17 TFs that were significantly upregulated in TNBCs and focused on delineating the role of Engrailed-1 (EN1), a neural-specific TF. They found that downregulation of EN1 in TNBC cell lines significantly reduced viability and tumorigenicity, as well as affected the expression of genes involved in WNT and Hedgehog signaling pathways [142]. Furthermore, high EN1 expression levels were associated with brain metastasis and poor OS. In a different study, EN1 expression was associated with unfavorable OS in QNBC patients. It has been proposed that EN1 may promote the proliferation, migration, and multinucleation of QNBC cells via the transcriptional activation of HDAC8, UTP11L, and ZIC3 [143]. In the same study, the ability of actinomycin to inhibit EN1 was also shown. Recently, multi-functionalized nanoparticles have been formulated to specifically target EN1 with less toxicity; these await further testing in clinical trials [144]. Notably, Peluffo G et al. identified the TF CEBPB, peroxisome proliferator-activated receptor delta (PPARD), and thyroid hormone receptor-interacting protein 13 (TRIP13) as promising potential biomarkers for QNBC [142].

### SKP2

As part of the SCF-SKP2 ubiquitin ligase complex, S-phase kinase-associated protein 2 (SKP2) is involved in the degradation of p21, p27 (a CDK inhibitor), and p57, among other proteins. SKP2 is a known oncoprotein being involved in DNA replication during the S-phase of the cell cycle [145]. SKP2 was identified as one of the key genes upregulated in TNBC compared to non-TNBC samples [103]. Although the impact of the differential expression of SKP2 between QNBC and TNBC remains unclear, AR was identified as an upstream regulator of SKP2 in prostate cancer cells. Additionally, AR expression levels were negatively correlated with SKP2 expression levels, and SKP2 overexpression resulted in reduced AR expression and activity [139, 146–150]. The association between SKP2 and negative AR status underpins SKP2 as a potential therapeutic target in QNBC. A small molecular inhibitor is currently being developed to inhibit SKP2-p26 interaction and subsequent p27 degradation [151].

## Conclusions: future perspectives and implementation

QNBC is an aggressive and poorly understood form of BC. This review presents recent findings that aimed to improve our understanding of QNBC tumor biology required to identify prognostic markers and therapeutic targets. Studies assessing the differential expression of biomarkers between QNBCs and TNBCs may yield novel actionable targets unique to QNBC. These discoveries may also benefit TNBC patients of African ancestry, who are predominantly diagnosed with a QNBC phenotype. Cumulative evidence suggests that QNBC is highly proliferative and immunogenic, rendering it an ideal candidate for cytotoxic chemotherapy and immunotherapy. However, QNBCs have worse clinical outcomes even after treatment with adjuvant chemotherapy. Besides, acquired resistance to taxanes is also commonly observed. Thus, combining these approaches with agents targeting QNBC biomarkers may enhance treatment response and improve prognosis.

A vast majority of these markers can be evaluated through clinically applicable methods such as immunohistochemistry (IHC). However, the successful characterization of QNBC patients and tailored clinical decision-making required the establishment of robust AR expression cutoffs. Although nuclear staining indicates active AR signaling, there is currently no standard scoring method for nuclear AR IHC staining. Often, CAP/ASCO staining guidelines for ER and PR are applied for AR since they stain similarly; however, there is no consensus on the threshold value for AR status assessment. One way to overcome this is to measure AR levels using a more reliable androgen-driven gene signature. Determining prior treatment, clinical outcomes, and key AR-dependent proteins, which may differ according to BC subtypes, will shed more light on the mechanistic action and prognostic value of AR in TNBC required for its implementation into routine clinical testing.

It is imperative to treat QNBC as a unique disease and thoroughly investigate its biology. Examining the role of epidemiological and non-biological factors in QNBC is equally important in gaining a holistic understanding of its etiology and uncovering novel modifiable risk factors.

## Data Availability

NA
